# Identification of a Patient Population Previously Not Considered for Organ Donation

**DOI:** 10.7759/cureus.805

**Published:** 2016-09-26

**Authors:** Brad Barrois

**Affiliations:** 1 Quality, Louisiana Organ Procurement Agency

**Keywords:** organ donation, kidney transplant

## Abstract

For the foreseeable future, more individuals will need a kidney than there are kidneys available for transplant. This is not a new issue, and it is one that will not likely be solved anytime soon. While recent initiatives have focused on efficiently allocating kidneys in order to maximize supply, a shortage will remain.

Currently, organs are made available for transplant through three different processes: donation after brain death declaration (BD), donation after circulatory death (DCD), and living donation (one healthy individual donates to a person in need). The objective of this article is to discuss the possibility of a fourth option in imminent death single kidney donation (IDSKD) and its potential effects on the future of donation and transplantation. During our study, IDSKD had the potential to increase the number of kidneys transplanted in our service area by approximately 5%.

## Introduction

The purpose of this study was to identify an alternative group of patients who were unable to become an organ donor after brain death declaration or donation after circulatory death. The primary reason these patients were unable to donate is due to intact neurological responses, which typically would prevent the patient from cardiac arresting within a given timeframe. All patients who were focused on during the study were referred to the Louisiana Organ Procurement Agency (LOPA) due to being hospital-bound, ventilator dependent, and their families had made the decision to terminally extubate. 

Imminent death single kidney donation (IDSKD) would allow a patient population who currently are deemed unsuitable for organ donation to become a donor. Once the patient’s family has decided to terminally extubate, the patient would be evaluated for donation after circulatory death (DCD). If the organ procurement organization (OPO) determines that DCD is not an option, then the patient could be evaluated for IDSKD. This process allows a family to authorize the removal of a single kidney from the potential organ donor. If the family approves of this option, the OPO and hospital would complete all legally required paperwork and required testing, per the United Network for Organ Sharing (UNOS) policy. Once a recipient is identified, the donor would be brought to the operating room, and a nephrectomy would be performed by the recipient’s transplant surgeon. Once the surgery is complete, the donor would then be brought back to the ICU, or designated area. The family’s decision to terminally extubate the patient could then be carried out by the appropriate hospital staff.

While this process is not an original thought, addressing the issue of an eligible patient population is. To date, no known long-term study has been completed to assess the potential for this type of organ donation. 

## Materials and methods

This study focused on data collected from 2014-2015. The original use of the data used in this study was not intended for this project. This study is a byproduct of research currently being completed by the Louisiana Organ Procurement Agency (LOPA) attempting to accurately predict the likelihood a patient would cardiac arrest in a timely manner in order to facilitate DCD. In order for donation after circulatory death to take place, the potential donor must cardiac arrest within two hours. Outside of the allotted time, the potential donor is no longer eligible for organ donation. The original research evaluated numerous patient demographics including, but not limited to, age, body mass index (BMI), cause of death, intact neurological responses, and various lab values. Patients would be evaluated once the LOPA was informed of a family’s decision to terminally extubate a patient who was ventilator dependent and hospital-bound. The LOPA clinical staff were responsible for the initial data collection and evaluation.

**Figure 1:**
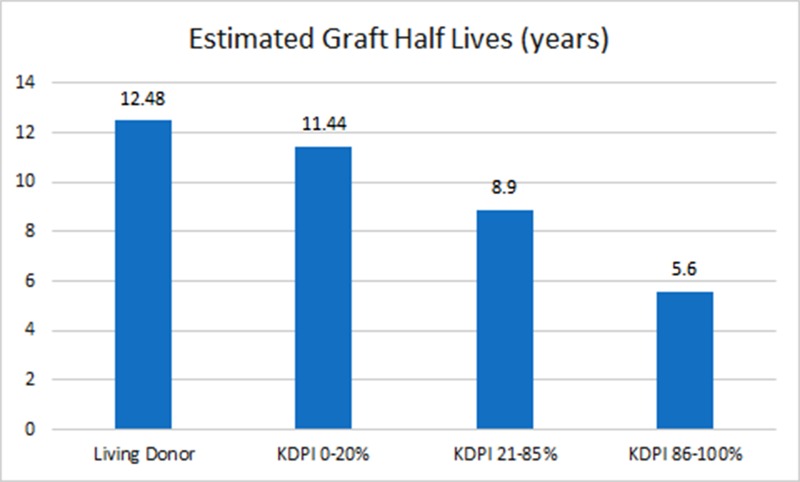
Estimated Graft Half Lives (years)

Under the advisement of the LOPA’s medical director, additional metrics would be necessary to properly assess the potential donor pool for IDSKD, primarily a kidney donor profile index (KDPI), history of diabetes, and the patient’s age. A patient’s  KDPI is determined using the KDPI calculator provided by the Organ Procurement and Transplantation Network (OPTN). Variables used in the calculation are as follows: age, height, weight, ethnicity, history of hypertension, history of diabetes, cause of death, serum creatinine, as well as Hepatitis C status. Once all information is entered, the calculator provides a percentage. According to OPTN, “lower KDPI score is associated with longer estimated function, while higher KDPI scores are associated with shorter estimated function. For example, a kidney with a KDPI of 20% is expected to have shorter longevity than 20% of recovered kidneys (i.e., longer function than 80% of recovered kidneys)” [1]. Figure 1 compares estimated graft half-lives (years) of a living donor to a deceased donor as the KDPI increases [1].

Since IDSKD is neither a deceased donor nor is it a traditional living donor, a metric, such as a KDPI, was needed for evaluation. Given that it provides an estimation of graft function, it was determined to be a pertinent metric for evaluating the donor population for IDSKD. Traditionally, a potential donor’s KDPI is only calculated in a deceased donation scenario, not for living donors. In imminent death single kidney donation, the patient does not fit the definition for either type of donation, making the use of KDPI unique for the current study.

Calculation of the potential donor’s KDPI was completed by the LOPA’s Data Manager. All necessary data needed for completion of the KDPI calculation were obtained after a potential donor was referred to the LOPA. 

Once data collection was completed, it then needed to be filtered according to the LOPA’s medical director’s criteria. The following criteria were used as filters: patients over the age of 60 were removed, those patients that had become a donor, those declared brain dead, history of diabetes, a KDPI above 85%, or if the family authorized for donation but the process was stopped due positive serological results, poor organ function, or inability to locate a potential recipient.

## Results

Twenty-five potential imminent death single kidney donors were identified. These patients' ages ranged from 14 to 59 years of age, with a median age of 45 and mean of 44. Calculated KDPI’s ranged from 15% to 84%, with a median of 59% and a mean of 56%.

Table 1 displays the identified potential donors with their corresponding KDPI as well as age. The list is sorted by descending age.

**Table 1:** Potential Donors with Corresponding KDPI and Age Potential donors are listed in descending order by age.

Case #	KDPI	Age
14812180513	84%	59
15127212817	83%	59
14504174134	70%	56
14606125758	63%	56
14718110423	83%	56
14728081000	73%	55
14322042142	70%	54
14402082732	52%	54
14902033139	59%	52
14318010316	30%	50
14819060522	57%	46
15116151900	82%	46
14409004803	45%	45
14715102508	45%	44
15222000521	55%	43
14501125747	77%	42
15224110146	59%	42
15120100409	39%	41
14722060926	62%	38
15130183944	61%	33
15621053651	30%	33
14506202425	18%	30
15602034535	15%	26
14818065039	51%	18
15430121514	32%	14

During the data collection time period, the LOPA transplanted 483 kidneys. The additional 25 possible IDSKD would have accounted for an increase of 5.17% kidneys transplanted. During this same collection period, 23,820 kidneys were transplanted nationally. A 5% increase nationally could have yielded an estimated 1,191 live-saving transplantable kidneys.

The patient population on whom this study was focused were referred to the LOPA during their most current hospital admittance. Average times from admittance to extubation ranged from 10 hours to 690 hours. The average time from admittance to extubation was 140.8 hours, or 5.8 days. There was no correlation between the length of time to extubation and an increase in a patient’s KDPI. Extubation time for case #14506202425 was not readily available, therefore it was not included in the average calculation.

## Discussion

At this time, the LOPA is formulating an implementation strategy for IDSKD. The most likely scenario for implementation will require a trial period at a local transplant facility. The LOPA would initially evaluate a patient's suitability for donation after circulatory death. If the patient does not meet the criteria, they would then be evaluated for IDSKD. By trialing IDSKD in a local transplant facility, it would allow greater control of the possible variables involved until the process is perfected. Educating and preparing the hospital staff would be considered paramount during this trial period.

As preparation continues, the OPO industry is faced with a primary issue. The United Network for Organ Sharing (UNOS) currently does not recognize imminent death single kidney donation. Therefore, there are no policies in place governing recovery and allocation of recovered kidneys through this process. Additional communication and process formalization with UNOS will be required to move forward in the future.

IDSKD will undoubtedly bring in ethical questions. An anticipated concern is hastening the death of the donor due to the removal of a kidney. According to the National Kidney Foundation, “Living donation does not change life expectancy, and does not appear to increase the risk of kidney failure. In general, most people with a single normal kidney have few or no problem...” [2]. While IDSKD is not technically considered living donation under its formal definition, the risks involved in the two processes have their similarities. 

We realize limitations may exist by using the Kidney Donor Profile Index. Primarily, the fact that it does not calculate how well a kidney will actually function once transplanted or just how well it might outperform other kidneys transplanted based on historical data. Fortunately, our goal was not to determine the functionality of the kidneys once transplanted. The goal was to identify a group of patients who, based on criteria provided by our medical director, would possibly be considered suitable for donation. Using the KDPI allowed us to take into account many different patient demographics and provided a metric on which we would be able to filter results. Ultimately, each transplant facility would be responsible for determining their own criteria for acceptance of a kidney based on the information provided to them.

Another task that will need to be addressed lies in how OPOs will be judged by governing agencies, primarily the federal government. Currently, OPOs are judged based on metrics that rate how productive and efficiently they are able to maximize organs from a potential donor. IDSKD would have a direct and negative impact on their efficiency rating.

## Conclusions

While it is clearly evident that imminent death single kidney donation will not completely address the entire shortage of kidneys the nation is currently facing, it does have the potential to provide more life-saving transplantable kidneys to patients in need. Ultimately, our responsibility as the organ procurement and transplant community is to ensure that those in need of a transplant are provided with said opportunity. We have a responsibility to be innovative in pursuing all legal and ethical options to ensure that no person dies while waiting for a transplant.
